# A Comparative Evaluation of Efficacy of *Streptococcus mutans* Counts in Saliva: An *in vivo* Study

**DOI:** 10.5005/jp-journals-10005-1492

**Published:** 2018-04-01

**Authors:** Megha Sharma, Inder K Pandit, Nikhil Srivastava, Neeraj Gugnani, Monika Gupta

**Affiliations:** 1 Senior Resident, Department of Dental Surgery, Vardhman Mahavir Medical College & Safdarjung Hospital, New Delhi, India; 2 Principal, Professor and Head, Department of Pedodontics and Preventive Dentistry, D.A.V. (C) Dental College & Hospital, Yamunanagar, Haryana, India; 3 Principal, Professor and Head, Department of Pedodontics and Preventive Dentistry, Subharti Dental College, Meerut, Uttar Pradesh, India; 4 Professor, Department of Pedodontics and Preventive Dentistry, D.A.V. (C) Dental College & Hospital, Yamunanagar, Haryana, India; 5 Professor, Department of Pedodontics and Preventive Dentistry, D.A.V. (C) Dental College & Hospital, Yamunanagar, Haryana, India

**Keywords:** Chlorhexidine mouthwash, Dental caries, Fluor protector varnish, Probiotic (Yakult) and dentifrice, *Streptococcus mutans.*

## Abstract

Dental caries is a disease of multifactorial origin and *Streptococcus mutans* is considered as the chief pathogen responsible for its development. However, reduction in the number of pathogenic bacteria, particularly S. *mutans,* with the use of various preventive measures, can reduce dental caries to a significant level. Therefore, the present clinical study was undertaken to evaluate and compare the efficacy of toothbrushing, fluoride varnish, chlorhexidine mouthwash, and a probiotic (Yakult) in reducing the S. *mutans* counts in the saliva using Dentocult SM Strip Mutans kit in children. A total of 40 school students between the age group of 4 and 8 years were selected for the study and S. *mutans* count was taken on the first dental visit using Dentocult SM Strip Mutans test kit. After the initial scores of S. *mutans* were obtained, the children were divided into four groups and provided with different caries preventive regimen for 2 weeks. After 2 weeks, the scores of *S. mutans* were reevaluated for reduction in their counts, if any. The data thus collected were tabulated and statistically analyzed. All the groups showed a significant reduction in S. *mutans* counts with the highest reduction from the Fluor Protector varnish followed by chlorhexidine mouthwash, probiotic (Yakult), and toothbrushing.

**How to cite this article:** Sharma M, Pandit IK, Srivastava N, Gugnani N, Gupta M. A Comparative Evaluation of Efficacy of *Streptococcus mutans* Counts in Saliva: An *in vivo* Study. Int J Clin Pediatr Dent 2018;11(2):94-99.

## INTRODUCTION

Dental caries is a complex disease that is expressed as an interaction of various factors including the host, agent, substrate, and time.^1^ This concept of interplay of these factors is widely accepted and is aptly described by WD Miller’s chemicoparasitic theory, which postulates that the initial demineralization of tooth enamel is brought about by acids produced by bacteria present on the tooth.

From the day there has been pain associated with caries, scientists from all over have been finding ways not only to cure the already established lesion, but also to prevent it at an early age. The various caries preventive measures have been broadly classified into mechanical, chemical, and dietary control measures. Among the mechanical means, toothbrushing is the most common devise used for oral hygiene maintenance.^2^ Also, the bisbiguanide chlorhexidine, which has been studied extensively for over 20 years, is currently the most potent chemotherapeutic agent against mutans streptococci and dental caries.^3^

Furthermore, fluoride has been found to be the most effective cariostatic agent in the field of dentistry, especially in pediatric dentistry.^4^ There are various modes of fluoride application, and, among them, fluoride varnishes are known to cause most significant reduction in *S. mutans* counts, are recommended for use in preschool children, and seem to be the most suitable and documented regimen for the infants.

Prevention has gone one step ahead with a rapidly expanding arena of probiotics. They are live microbial food or feed supplements, which beneficially affect the host by improving its intestinal microbial balance and therefore, its nutritional health and well-being. Yakult, a probiotic has been commonly used for the gut, which contains *Lactobacillus casei* Shirota strain. A study by Hor et al^5^ showed that Yakult is a promising innovative therapy for caries prevention among children.

Various parameters have been used in the past to evaluate the efficacy of preventive measures. Microbial monitoring has been considered as an effective method for evaluating current caries activity and future caries risk.^4^ Reduction in the number of pathogenic bacteria, particularly *S. mutans,* can reduce dental caries to a significant level with the use of various preventive measures.

The application of microbial tests to assess caries risk in children was demonstrated initially by Krasse.^6^ In the first epidemiological surveys, tongue blades and agar plates were used, but these proved impractical for fieldwork and were replaced with liquid media into which the saliva-contaminated strips were inserted.^7^ These chair-side caries activity tests have been thoroughly compared with conventional selective agar plate culture. The comparison yields a good correlation with regard to detection of mutans streptococci.^8^

The Dentocult SM Strip Mutans test kit, introduced by Jensen and Bratthall,^9^ is a reliable method for measuring the status of dental caries in preschool children and also a valuable tool in the prevention and treatment of dental caries according to Shi et al.^10^ This is a chair-side caries activity test ensuring greater patient compliance, especially for young subjects, is less time consuming, needs minimum armamentarium, and facilitates sample selection.

The present clinical study was undertaken to evaluate and compare the efficacy of toothbrushing, fluoride varnish, chlorhexidine mouthwash, and a probiotic (Yakult) in reducing the *S. mutans* counts in the saliva using Dentocult SM Strip Mutans test kit in children.

## MATERIALS AND METHODS

The present study was conducted in the outpatient Department of Pedodontics and Preventive Dentistry, D.A.V. (C) Dental College & Hospital, Yamuna Nagar, Haryana, India, to evaluate and compare the efficacy of various caries preventive measures in reducing *S. mutans* counts in saliva using Dentocult SM Strip Mutans test kit.

An ethical committee approval was taken from the college authorities for conducting the study which was followed by a written consent from the parents of the school students selected as subjects regarding the commencement of the procedure.

A total of 40 school students between the age group of 4 and 8 years, having a decayed, missing and filled teeth score >5 were selected from various schools of Yamuna Nagar.

*Streptococcus mutans* count was taken for all the selected 40 students on the first dental visit using Den-tocult SM Strip Mutans test kit.

**Figs 1A to D:**
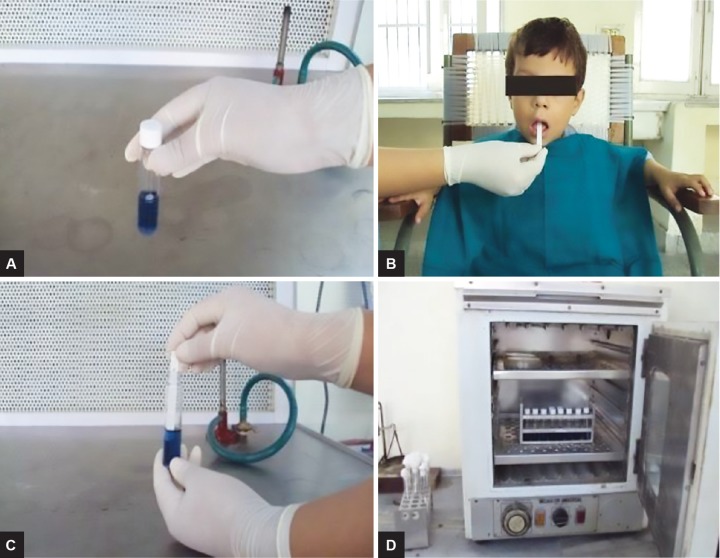
The procedure of Dentocult SM Strip Mutans test. (A) Bacitracin disks in the culture vials. (B) Rough-tipped strip pressed against patients tongue. (C) Strip placed in activated culture vial. (D) Vials incubated at 37°C for 48 hour

For the baseline status of *S. mutans,* the students were comfortably seated on the chair and unstimulated mid-morning salivary samples were collected. The baci-tracin disks provided in the Dentocult SM Strip Mutans test kit were placed in the selective culture broth about 15 minutes before sampling (Fig. 1A). Instructions were given to the patients to swallow any excess saliva and then the rough surface of the round tipped strip was pressed against the saliva remaining on the tongue (Fig. 1B). The strips were then placed in the activated culture vials (Fig. 1C) followed by their incubation in an upright position at 37°C for 48 hours (Fig. 1D).

**Fig. 2:**
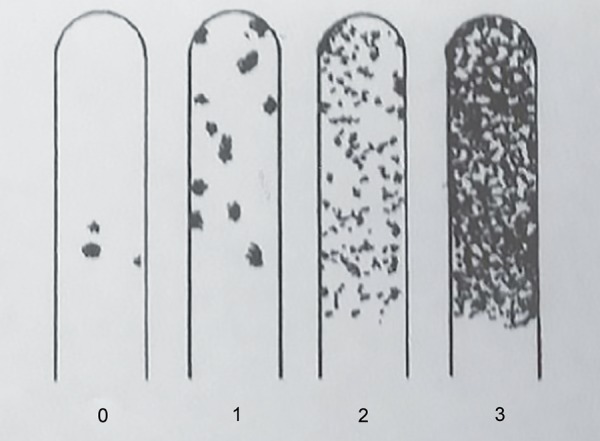
Model chart. Class 0: <10,000 CFU/mL; class 1: <100,000 CFU/mL; class 2: 100,000-1,000,000 CFU/mL; class 3: >100,000 CFU/mL; CFU: Colony-forming unit

After incubation, the presence of mutans streptococci was evidenced by dark blue to light blue raised colonies on the rough surface of the strip. Colonies suspended in the selective culture broth were excluded from evaluation. The pretreatment levels of *S. mutans* were then evaluated by comparing the colony density against a chart provided by the manufacturer (Fig. 2).

Inspection of the growth was done sideways against light or with a magnifying glass to look for raised colonies.

Following the assessment of the initial *S. mutans* counts, the school students were randomly divided into four equal groups and were provided with a caries preventive regimen for 2 weeks (Table 1).

After 2 weeks, reduction, if any, in the *S. mutans* counts was assessed using Dentocult SM Strip Mutans test. The strips were then placed with the smooth surfaces clipped and attached to the cap in the selective culture vial that were prelabeled with the names of the children. After the collection of samples in the respective vials, the names were blocked by a different person and a new code was given to each vial. The evaluator was completely blind with this coding procedure. The coded culture vials were incubated at 37°C for 48 hours. After 48 hours, the posttreatment *S. mutans* counts of all the subjects were available to the interpreter for evaluation of results. After the results were evaluated, the vials were decoded and the results were tabulated.

The pretreatment and posttreatment scores of each subject in all the four groups were compared for the evaluation of *S. mutans* counts after 2 weeks’ intervention of all the four caries preventive measures.

## RESULTS

**Graph 1:**
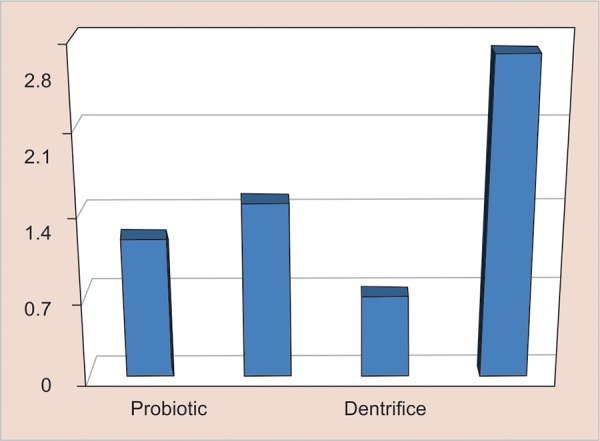
Overall reduction in S. *mutans* counts after 2 weeks of preventive regimes

Table 2 (Graph 1) shows the mean values of overall reduction in *S. mutans* counts after 2 weeks of exposure to respective caries preventive measures. Out of the four preventive measures, the mean difference for the dentifrice group was observed to be minimum (0.700 ± 0.6749) followed by probiotic (1.200 ± 0.6325) and chlorhexidine (1.500 ± 0.5270). Maximum reduction was observed in subjects in whom varnish was used as the preventive measure (2.700 ± 0.4830).

Table 3 shows one-way analysis of variance and Kruskal-Wallis test for the reduction in *S. mutans* counts was done using various preventive regimes, and highly significant differences (p < 0.05) on various inter- and intragroup comparisons were observed.

**Table 1:** **Table 1:** Distribution of samples and their preventive regimes

*Group number*		*Caries preventive measure*		*Regimen of the preventive measure (for 2 weeks)*		*Number of samples*	
Group I		Probiotic milk (Yakult)		Daily regimen of 65 mL bottle of Probiotic milk (Yakult®)		10	
Group II		Chlorhexidine mouthwash (Hexidine®)		Daily regimen of chlorhexidine mouthwash (Hexidine®) two times a day		10	
Group III		Dentifrice (Babool)		Brushing twice daily with a nonfluoridated toothpaste (Babool)		10	
Group IV		Fluoride varnish (Fluor Protector)		Fluoride varnish (Fluor Protector) was applied on the initial visit			

**Table 2:** **Table 2:** Difference in *S mutans* counts following 2 weeks of different caries preventive measures

*Caries preventive measure*		*n*		*Mean*		*SD*		*SE*	
Probiotic milk (Group I)		10		1.200		0.6325		0.200	
Chlorhexidine (Group II)		10		1.500		0.5270		0.166	
Dentifrice (Group III)		10		0.700		0.6749		0.213	
Varnish (Group IV)		10		2.700		0.4830		0.152	

SD: Standard deviation; SE: Standard error

**Table 3:** **Table 3:** Analysis of variance (Kruskal-Wallis test) for efficacy of various caries preventive measures

*Preventive measure*		*n*		*Rank*		*Chi-square*		*df*		*p-value*		*Inference*	
Probiotic milk		10		16.65		24.101		3		0		HS	
(Group I)													
Chlorhexidine		10		20.50									
(Group II)													
Dentifrice		10		10.70									
(Group III)													
Varnish		10		34.15									
(Group IV)													

S: Significant at p < 0.05; NS: Not significant when p > 0.05, HS: Highly significant when p < 0.01, df: degree of freedom

Table 4 depicts nonsignificant results when group I (probiotic milk) was compared with group II (0.282) and group III (0.102) where Mann-Whitney test was used for intergroup comparisons. All other comparisons showed highly significant results. Highly significant results were observed when group IV was compared with group I (0.000), group III (0.000), and group II (0.001). Similarly, intergroup comparison of groups II and III also showed results of high significance (0.013).

## DISCUSSION

In the present clinical study, for the purpose of evaluating the reduction in the *S. mutans* counts in respective groups for various intergroup comparisons, the mean reduction scores were calculated for all the four groups. The mean reduction in *S. mutans* counts was significant in all the four groups in our study. This indicated that all preventive measures used in our study had the potential to reduce *S. mutans* counts.

In the present study, the probiotic group showed significant reduction in *S. mutans* counts. The *L. casie* Shirota strain in the probiotic (Yakult) might have replaced the normal commensals in the oral biofilm that might have contributed to significant reduction in the *S. mutans* scores. These results were in accordance with a study by Hor et al,^5^ where a decreased mutans streptococci count was observed following a 2-week consumption of Yakult, although the decrease was found to be nonsignificant. However, Hor et al^5^ conducted their study in adults with the age group of 21 to 40 years, while the present study was done on children. Therefore, the difference in oral ecology of child and adult might have contributed to a nonsignificant reduction in the previous study.

A significant mean reduction of *S. mutans* scores was also observed with chlorhexidine mouthwash.

**Table 4:** **Table 4:** Intergroup comparison of various caries preventive measures after 2 weeks using Mann-Whitney U test

*Intergroup comparison*		*Mann-Whitney U test*		*p-value*		*Inference*	
Group I *vs* II		–1.076		0.282		NS	
Group I *vs* III		–1.636		0.102		NS	
Group I *vs* IV		–3.615		0.000		HS	
Group II *vs* III		–2.471		0.013		HS	
Group II *vs* IV		–3.425		0.001		HS	
Group III *vs* IV		–3.806		0.000		HS	

S: Significant at p < 0.05; NS: Not significant, when p > 0.05; HS: Highly significant when p < 0.01

In the present study, 0.2% chlorhexidine mouthwash had exhibited a broad spectrum activity against *S. mutans* by preventing their colonization and reducing their number in the saliva. These results were similar to a study by Sari and Birinci,^11^ in which it was reported that patients using 0.2% chlorhexidine gluconate mouthrinse after toothbrushing every day once in the morning after breakfast and once in the evening before bedtime showed a significant decrease in *S. mutans* levels. These findings confirm those of Beyth et al^12^ in which chlorhexidine varnish was used.

Although significant, least mean reduction in *S. mutans* counts as compared with all the other three groups was observed in group III (dentifrice).

Toothbrushing is probably the most commonly performed oral hygiene practice in the world. The major purpose of this procedure is to lower the organisms in dental plaque that might be responsible for oral diseases/ conditions including dental caries, periodontal diseases, and halitosis.^13^ This mechanical cleansing effect might be responsible for the reduction in the *S. mutans* counts following toothbrushing regimen in the present study. Also, it could be due to ”Hawthorne effect”^14^ or a positive change in the behavior of subject as a result of special attention and status received from participation in an investigation.

Similar findings have been reported by Seow et al^15^ in which a single dental health education session and tooth-brushing instruction to mothers resulted in approximately a 25% reduction in mutans streptococci infection in young children from a relatively high socioeconomic status.

The minimum reduction in *S. mutans* following tooth-brushing in the present study could probably be because in this age group, there is decreased development of motor skills, difficulty in cleaning the teeth during the mixed dentition period leading to inadequate plaque removal from the caries-prone sites and also, according to Svan-berg,^16^ sometimes toothbrushes are shown to be heavily infected by *S. mutans,* which may lead to the spread of these and other organisms from one site to another.

The maximum mean reduction in *S. mutans* counts was observed in group IV where varnish was used as a preventive measure (2.700 ± 0.4830).

The high mean reduction in the *S. mutans* counts with the use of Fluor Protector varnish has been supported by a large number of studies conducted.^17,18^

The reduction of bacterial counts following the use of Fluor Protector varnish could be due to increase in the depth of fluoride penetration in enamel and root surfaces and enhanced retention of surface fluoride^19^ and increased contact time between fluoride and enamel.^20^ The varnish also exhibits antibacterial effect by fluoride leached out and taken up by the tooth to inhibit the bacterial growth or direct entry of fluoride into the bacterial cell and inhibition of various cellular processes.^4^

The results of the present study are in accordance with the study conducted by Jeevarathan et al,^4^ in which statistically significant reduction (p = 0.000) in *S. mutans* counts was observed in plaque samples in 24 hours following the application of Fluor Protector varnish. Munshi et al^18^ evaluated the demineralizing inhibitory and antibacterial effects of Fluor Protector in an *in vitro* study and found that it had highest demineralizing inhibitory effect and the lowest antibacterial effect when compared with Bifluoride-12 and Fluoritop SR, which was due to a greater uptake of fluoride by enamel in the tooth sections treated by Fluor Protector in spite of its lower fluoride content.^21^

Nonsignificant results were observed when group I (probiotic) was compared with group II (chlorhexidine). The nonsignificant difference among the groups might be due to the fact that both of them have a similar potential of “washing off” the cariogenic bacteria, that is, probiotic replaces the normal habitat in the oral biofilm and chlorhexidine, being bacteriostatic, prevents bacterial colonization on the tooth surface. Therefore, almost similar effects might have resulted in a nonsignificant difference between the two when intergroup comparisons were made. However, the mean reduction of both the groups showed highly significant results.

Highly significant results were obtained when Fluor Protector varnish was compared with probiotics. The greater reduction of Fluor Protector as compared with probiotic could be attributed to multiple effects exhibited by the varnish in *S. mutans* reduction and caries inhibition as compared with probiotics. Probiotics act by a single mechanism of displacing off the pathogenic bacteria from the biofilm by competitive inhibition, whereas Fluor Protector varnish may increase the depth of fluoride penetration in enamel and root surfaces, and enhances retention of surface fluoride,^19^ increases the contact time between fluoride and enamel,^20^ and exhibits its antibacterial effect by fluoride being leached out and taken up by the tooth to inhibit the bacterial growth or direct entry of fluoride into the bacterial cell and inhibition of various cellular processes.^4^ Also, highly significant results were observed in the present study when dentifrices were compared with chlorhexidine mouthwash and Fluor Protector varnishes.

The decreased reduction of dentifrice group as compared with the chlorhexidine and varnish in the present study could be attributed to the fact that toothbrushing is not always carried out thoroughly, does not remove plaque from some of the caries-prone sites and is shown sometimes to be heavily infected with *S. mutans.^16^* The reduction observed in toothbrushing in the present study would only have been due to the “Hawthorne effect” or participation effect, which would have influenced the oral hygiene maintenance of the subjects.

However, when intergroup comparisons were made between Fluor Protector group and chlorhexidine group, there was a greater reduction with the Fluor Protector group as compared with the chlorhexidine group.

The decreased reduction following chlorhexidine mouthwash as compared with the varnish appears to be related primarily to incomplete eradication of mutans streptococci following the chlorhexidine mouthwash regimen, rather than reinoculation with the pathogen.^21^ Also, not all people harboring high mutans streptococci levels, however, respond optimally to hexidine treatment. Once the chlorhexidine treatment ceases, how quickly people return to pretreatment *S. mutans* levels varies considerably from subject to subject.^22^ Therefore, subject-to-subject variation and incomplete eradication following chlorhexidine regimen would have contributed to *S. mutans* reduction as compared with Fluor Protector varnish.

## CONCLUSION

All the four caries preventive measures were effective in significantly reducing the *S. mutans* counts in saliva. Although there was a reduction in *S. mutans* scores among all the four groups, highest reduction was observed following the use of Fluor Protector varnish, and brushing with a nonfluoridated dentifrice showed least reduction as compared with the other caries preventive measures. Based on the results of the present study, the efficacy of various caries preventive measures can be graded as follows:

Fluor Protector> Chlorhexidine mouthwash> Probiotic (Yakult)> Brushing with a nonfluoridated dentifrice
